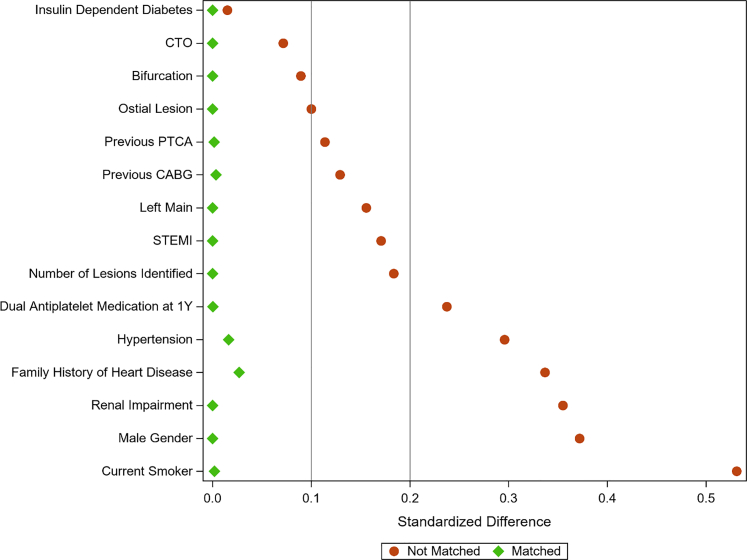# Prognosis of PCI in the Older Adult Population: Outcomes From the Multicenter Prospective e-ULTIMASTER Registry

**DOI:** 10.1016/j.jscai.2022.100442

**Published:** 2022-08-24

**Authors:** Majdi Saada, Ofer Kobo, Floris Kauer, Orazbek Sakhov, Peep Laanmets, Rajpal Abhaichand, Iñigo Lozano, Jim Crowley, Gurupreet Singh Wander, Mamas A. Mamas, Ariel Roguin, e-ULTIMASTER Investigators

**Affiliations:** a Hillel Yaffe Medical Center, Technion–Faculty of Medicine, Israel; b Department of Cardiology, Albert Schweitzer Ziekenhuis, Dordrecht, the Netherlands; c Department of Interventional Cardiology, City Heart Center, Almaty, Kazakhstan; d Department of Cardiology, North Estonia Medical Centre, Tallinn, Estonia; e Department of Cardiology, L.R.G. Naidu Cardiology Research Institute and Clinic, Kuppuswamy Naidu Memorial Hospital, Coimbatore, India; f Department of Cardiology, Hospital Cabueñes, Gijon, Spain; g Galway University Hospital, Galway, Ireland; h Hero DMC Heart Institute, Dayanand Medical College, Ludhiana, Punjab, India; i Keele Cardiovascular Research Group, Centre for Prognosis Research, Keele University, Keele, United Kingdom

**Keywords:** age, coronary artery disease, elderly, older adults, outcome, stent, vascular disease

## Abstract

**Background:**

Older adult patients undergoing percutaneous coronary intervention (PCI) are usually excluded from clinical trials. This study aimed to assess 1-year clinical outcomes in patients aged >80 years.

**Methods:**

This all-comer registry included patients who underwent PCI using the Ultimaster stent. Primary clinical endpoint was target lesion failure (TLF), a composite of cardiac death (CD), target vessel–related myocardial infarction (TV-MI), or clinically indicated target lesion revascularization (CD-TLR).

**Results:**

In total, 3286 (8.8%) patients aged ≥80 years were compared with 33,912 patients aged <80 years. The older adult patients included more women, had more comorbidities and exhibited more complex coronary anatomy. The incidence of TLF was higher in the older adult group (5.6% vs 3.0%, *P* < .0001), as well as for all-cause mortality (6.2% vs 1.7%, *P* < .0001), CD (3.3% vs 1.1%, *P* < .0001), and TV-MI (1.7% vs 0.8%, *P* < .0001), but not for CD-TLR (1.9% vs 1.7%, *P* = .15). After the inverse propensity score weighted analysis, aged ≥80 years was associated with increased risk of TLF (HR, 1.42; 95% CI, 1.22-1.66; *P* < .0001), CD (HR, 1.67; 95% CI, 1.136-2.06; *P* < .0001), and TV-MI (HR, 1.66; 95% CI, 1.24-2.24; *P* < .001) but not for CD-TLR (HR, 1.10; 95% CI, 0.85-1.43; *P* = .45).

**Conclusion:**

Older adult patients had a higher incidence of TLF, CD, and TV-MI but with no difference in the incidence of recurrent revascularization or stent thrombosis. Although PCI in older adults is relatively safe, higher rates of cardiac events should be considered.

## Highlights

• Age is an independent predictor of adverse outcome.
• Older adult patients are often excluded from clinical trials because of limited life expectancy.
• Older adults constitute a significant proportion (8.8%) of patients undergoing PCI.
• Older adult patients have an increased risk for death and MI but similar risk for recurrent revascularization and stent thrombosis.

## Central Illustration

**Figure:**
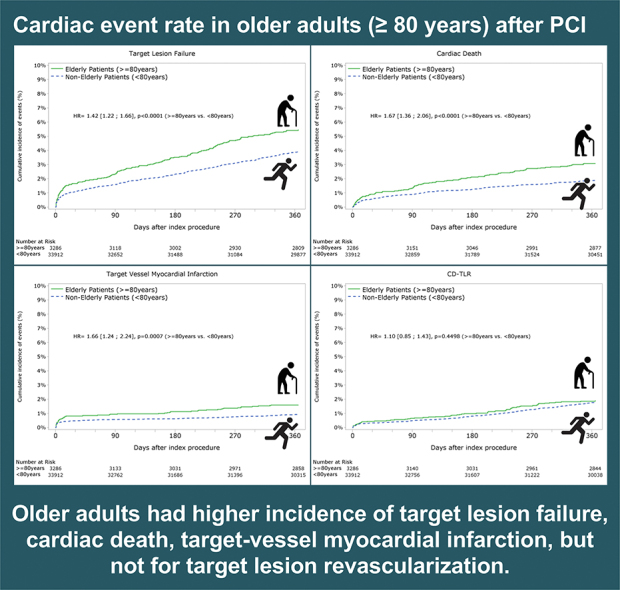
Cardiac event rates after PCI.

## Introduction

Coronary artery disease (CAD) is the most common cause of death worldwide and is a leading cause of mortality and morbidity in older adults.^1^, ^2^, ^3^, ^4^ Globally, the population is aging, thanks to the progress made in medical science. Moreover, the number of older adult patients with CAD undergoing percutaneous coronary intervention (PCI) increased over the past decade^5^, ^6^, ^7^ and is expected to grow even further.^8^

Prior studies have suggested that age is an independent predictor of adverse outcome after PCI.^9^^,^^10^ However, older adults aged >80 years are often excluded from major clinical trials of cardiovascular interventions because of concerns about the increased risk of adverse events and limited life expectancy.^11^ In addition, older patients with CAD are less likely than younger patients to undergo invasive revascularization even in current clinical practice.^12^, ^13^, ^14^ Therefore, knowledge regarding the long-term outcome of the older adults referred for PCI in the current era of improved techniques, devices, and pharmacotherapy is scarce. The few studies on this population mainly include patients with acute coronary syndrome, far fewer patients with stable CAD,^15^^,^^16^ and lack long-term outcomes.^15^, ^16^, ^17^ The reported outcomes after PCI in the older adults have differed widely.

This study aimed to investigate the characteristics and clinical outcomes in the older adults undergoing PCI in the contemporary drug-eluting stent (DES) era from one of the largest cohorts, including both acute and elective indications.

## Methods

The e-ULTIMASTER registry is an all-comer patient population with indications for PCI, which enrolled 37,198 patients. We analyzed the population according to age and compared the older adults (aged ≥80 years) with the nonolder adult (aged <80 years) patients. Inclusion and exclusion criteria^18^ were minimal to better evaluate the Ultimaster stent (Terumo Corporation). Sites in Europe, Asia, Africa, Middle East, South America, and Mexico participated in the registry (see Appendix for list of participating sites), using a thin-strut (80 μm) cobalt–chromium sirolimus eluting stent. This stent features a biodegradable polymer coating (poly-d,l-lactic acid polycaprolactone) that is fully metabolized through dl-lactide and caprolactone into carbon dioxide and water in 3 to 4 months. This coating is applied on the abluminal side of the struts only; after resorption, a bare-metal stent is obtained.

The primary end point was 1-year target lesion failure (TLF) defined as the composite of cardiac death, target vessel–related myocardial infarction (TV-MI), and clinically driven target lesion revascularization (CD-TLR). The patient-oriented composite end point was defined as all deaths, any myocardial infarction (MI), and any revascularization. Major adverse cardiac events were defined as cardiac death, any MI, and any revascularization. For MI, the extended historical MI definition was applied, which primarily uses creatine kinase myocardial band, or if not available troponin, as cardiac biomarker criterion. Major bleeding was defined as a Bleeding Academic Research Consortium 3 or 5 bleeding. All primary end point–related events were adjudicated by an independent clinical events committee. The study was approved by the ethical committees of the participating sites, and all patients provided written informed consent. The clinicaltrials.gov identifier is NCT02188355.

The follow-up was performed 3 months and 1 year after the index procedure. Patients were contacted by telephone or by a visit to the outpatient clinic. Relevant information on adverse events, that is, death, MI, re-PCI, coronary artery bypass graft, bleeding, vascular complication, or stent thrombosis, was collected.

### Statistical analysis

Continuous variables are presented as the mean ± SD and compared using the *t* test. Categorical variables are presented as frequencies (percentage) and compared using the χ^2^ test or Fisher exact test, as appropriate. The cumulative event rates were estimated by the Kaplan–Meier method and compared by the log-rank test. Hazard ratios (HRs) and 95% CIs were calculated using a Cox hazards regression analysis. An inverse propensity score weighted analysis was performed to address differences in baseline patient and lesion characteristics including the following 15 variables known to affect patient outcomes: current smoker, male sex, renal impairment, a family history of heart disease, hypertension, dual antiplatelet therapy at 1 year, the number of lesions identified, ST-elevation myocardial infarction, left main disease, previous coronary artery bypass grafting, previous PCI, ostial lesion, bifurcation lesion, chronic total occlusion, and insulin-dependent diabetes mellitus. A multinomial logistic regression model was performed to calculate the propensity score, predicting the probability of a patient being attributed to 1 of the 2 groups studied (older and younger patients) using the above-listed 15 baseline patient and lesion characteristics. The inverse of this propensity score (probability of belonging to the arm the patient was attributed to) was then used as weights in the weighted analyses and was calculated as 1/(propensity score). Standardized differences of variables used to generate the propensity score before and after inverse-weighted propensity score adjustment were calculated (Supplemental Figure S1). After adjustment, all covariates in the planned propensity score had weighted standardized differences <0.1, which indicates an equilibration of these covariates between the groups. Analyses were performed using SAS software, version 9.4 (SAS Institute Inc)**.**

## Results

**Figure 1:**
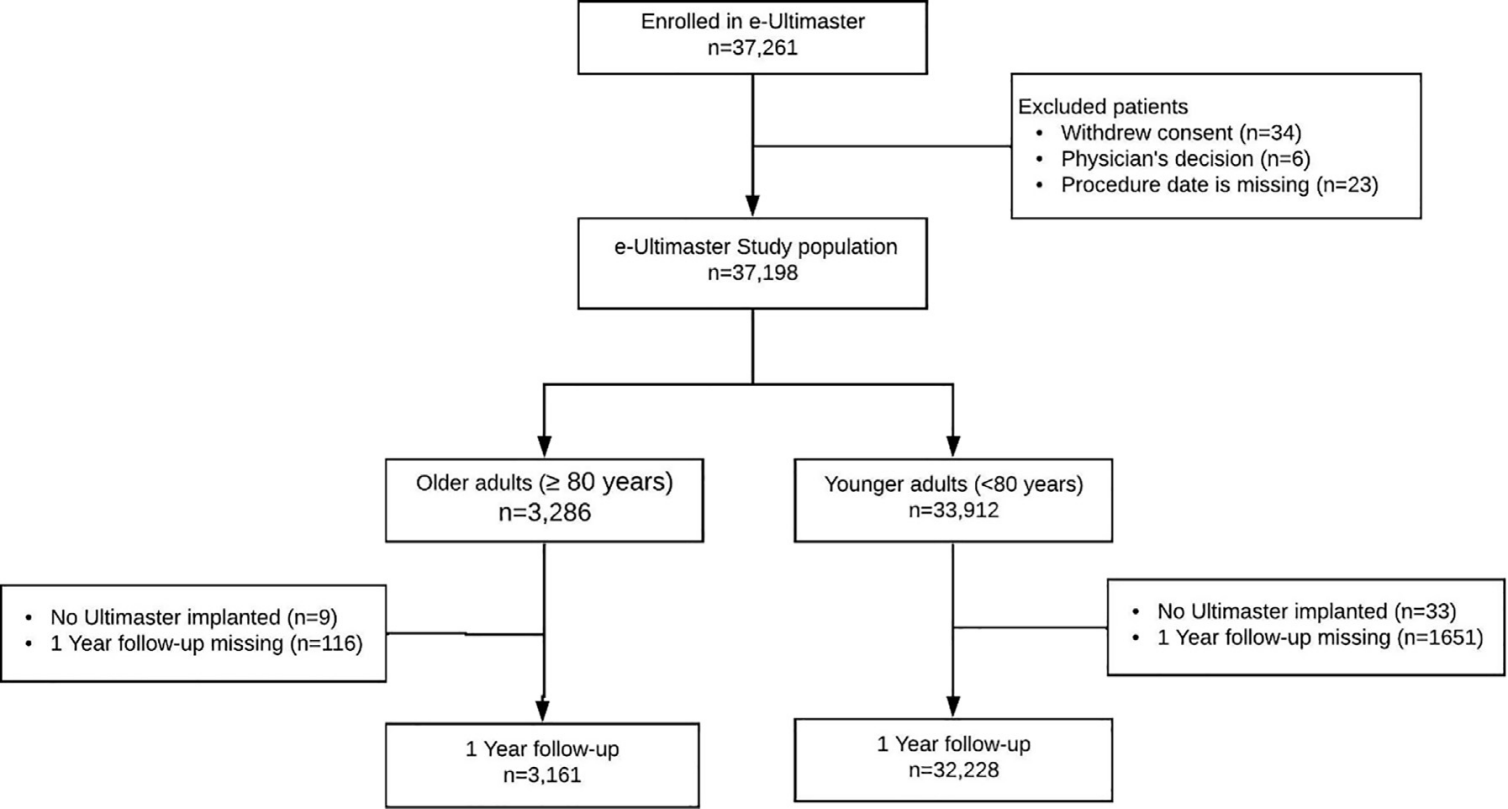
**Flowchart of the study.**

A total of 37,198 patients who underwent PCI were included in our analysis (Figure 1), of which 8.8% (N = 3286) were included in the older adult group. Baseline characteristics of these patients and procedural characteristics are summarized in Table 1. The mean age of the older adult group was 83.4 ± 3.1 years, compared with 62.3 ± 10.0 years in the younger group (*P* < .0001). Patients in the older adult group were more likely to be women (30.9% vs 22.5%, *P* < .0001) and had a greater prevalence of hypertension and renal disease than the younger patients (79.9% vs 66.6% and 18.1% vs 5.9%, respectively, both *P* < .0001), whereas they were less current smokers (5.2% vs 25.4%, *P* < .0001).

**Table 1:** Baseline patient and angiographic characteristics.

	Older adults (≥80 y)	Younger adults (<80 y)	*P*
	N = 3286	N = 33,912	
Male sex	60.1 (1976/3286)	77.5 (26,281/33,912)	<.0001
Age, y	83.4 ± 3.1 (3286)	62.3 ± 10.0 (33,912)	<.0001
Diabetes mellitus	29.4 (937/3189)	28.3 (9442/33,383)	.19
Insulin-dependent diabetes mellitus	5.5 (174/3189)	5.8 (1947/33,383)	<.0001
Non–insulin-dependent diabetes mellitus	23.9 (762/3189)	22.4 (7487/33,383)	.06
Current smoker	5.2 (154/2956)	25.4 (7743/30,524)	<.0001
Hypercholesterolemia	61.1 (1773/2900)	59.8 (17,689/29,579)	.16
Hypertension	79.9 (2402/3006)	66.6 (20,438/30,678)	<.0001
Positive family history	21.2 (355/1675)	37.5 (6904/18,406)	<.0001
Peripheral vascular disease	11.3 (341/3022)	6.2 (1914/30,858)	<.0001
Previous myocardial infarction	24.5 (747/3054)	22.7 (7105/31,369)	.02
Previous PCI	30.9 (957/3095)	25.5 (8069/31,592)	<.0001
Previous CABG	8.8 (273/3108)	5.3 (1665/31,454)	<.0001
Renal impairment	18.1 (575/3170)	5.9 (1973/33,237)	<.0001
Chronic coronary syndrome	46.4 (1524/3286)	44.7 (15,148/33,912)	.06
Acute coronary syndrome	53.5 (1757/3286)	55.3 (18,742/33,912)	.05
Unstable angina	10.4 (341/3286)	12.0 (4074/33,912)	.0056
NSTEMI	29.2 (959/3286)	22.5 (7618/33,912)	<.0001
STEMI	13.9 (457/3286)	20.8 (7050/33,912)	<.0001
STEMI with cardiogenic shock	0.2 (8/3286)	0.3 (113/33,912)	.39
Femoral arterial access	16.5 (541/3286)	17.4 (5912/33,912)	.16
Radial arterial access	81.0 (2660/3286)	80.6 (27,336/33,912)	.64
Femoral and radial arterial access	2.1 (70/3286)	1.5 (518/33,912)	.01
Left main	6.0 (198/3286)	2.8 (960/33,912)	<.0001
Right coronary artery	33.4 (1096/3286)	34.4 (11,669/33,912)	.22
Left anterior descendants	52.0 (1708/3286)	51.5 (17,469/33,912)	.61
Left circumflex	26.8 (881/3286)	27.9 (9462/33,912)	.18
Arterial or venous bypass graft	2.5 (83/3286)	1.1 (361/33,912)	<.0001
Any severe/moderate calcification	27.8 (914/3286)	18.2 (6160/33,912)	<.0001
Any in-stent restenosis	5.9 (194/3286)	5.2 (1753/33,912)	.07
Any ostial lesion	9.6 (315/3286)	6.7 (2275/33,912)	<.0001
Any bifurcation	14.7 (483/3286)	11.5 (3912/33,912)	<.0001
Any chronic total occlusion	3.7 (123/3286)	5.2 (1761/33,912)	<.001
Any long lesion (total stent length ≥25.0 mm)	39.4 (1296/3286)	41.7 (14,129/33,912)	.01
Any small vessel (any stent ≤2.25 mm)	11.1 (363/3286)	8.1 (2732/33,912)	<.0001
No. lesions treated	1.4 ± 0.7 (3285)	1.3 ± 0.6 (33,873)	<.0001
No. implanted stents	1.62 ± 0.90 (3282)	1.55 ± 0.86 (33,816)	<.0001
Total implanted stent length, mm	31.2 ± 19.9 (3272)	31.1 ±19.6 (33,760)	<.0001
Intravascular imaging	9.83 (257/2614)	8.34 (2162/25,933)	.01

Values are % (n/N) or mean ± SD.

CABG, coronary artery bypass grafting; NSTEMI, non-ST-elevation myocardial infarction; PCI, percutaneous coronary intervention; STEMI, ST-elevation myocardial infarction.

The angiographic characteristics were different between the groups. The older adult group had more lesions treated during the index procedure compared with the younger patients (1.4 ± 0.7 vs 1.3 ± 0.6, *P* < .0001), with higher rates of the left main coronary artery treatment (6.0% vs 2.8%, *P* < .0001), calcified lesions (27.8% vs 18.2%, *P* < .0001), bifurcation stenting (14.7% vs 11.5%, *P* < .0001), and ostial lesions (9.6% vs 6.7%, *P* < .0001). There were less chronic total occluded lesions (3.7% vs 5.2%, *P* < .0001) in the older adult group. The use of the radial artery approach was similarly in both groups (81.0% vs 80.6%, *P* = .64). At hospital discharge, 95.6% in the older adult group and 97.6% in the younger group were prescribed dual antiplatelet therapy (*P* < .0001). Aspirin was used by 95.8% and 97.7% of patients, respectively (*P* < .0001).

There were 35,389 (95.1%) patients with a 1-year follow-up (Figure 1). The major baseline characteristics of patients available for 1-year follow-up and missing patients are presented in Supplemental Table S1. The crude event rates at 1 year are tabulated in Table 2. The primary end point of TLF occurred in 5.6% in the older adult group compared with 3.0% in the younger group (*P* < .0001). All-cause mortality was significantly higher in the older adult group than in the younger group (6.2% vs 1.7%, *P* < .0001), as well as death from cardiac causes (3.3% vs 1.1%, *P* < .0001), all MI (2.3% vs 1.1%, *P* < .001), TV-MI (1.7% vs 0.8%, *P* < .001), and the patient-oriented composite end point (10.9% vs 6.1%, *P* < .0001). There were increased stroke incidences in the older adult group (0.4% vs 0.1%, *P* < .0001). More bleeding-related complications were observed among the older adult group than those in the younger group (1.6% vs 0.5%, *P* < .01). The intake of dual antiplatelet therapy at 1 year was less in the older adult group (62.7% vs 71.4%, *P* < .0001), but more patients were using an oral anticoagulant (12.8% vs 5.2%, *P* < .0001).

**Table 2:** Event rates at 1 year for the crude and the adjusted analysis for elderly and nonelderly patients.

	Crude analysis			Adjusted analysis		
	Older adults (≥80 y)	Younger adults (<80 y) N = 32,228	*P*	Older adults (≥80 y) N = 3161	Younger adults (<80 y) N = 32,228	*P*
	N = 3161					
TLF	5.6 (178)	3.0 (957)	<.0001	5.4 (172)	3.9 (1267)	<.0001
POCE	10.9 (343)	6.1 (1963)	<.0001	10.6 (334)	7.4 (2385)	<.0001
MACE	6.8 (215)	3.8 (1215)	<.0001	6.6 (210)	4.7 (1518)	<.0001
All death	6.2 (196)	1.7 (550)	<.0001	5.9 (186)	2.9 (949)	<.0001
Cardiac death	3.3 (102)	1.1 (353)	<.0001	3.1 (97)	1.9 (618)	<.0001
All myocardial infarction	2.3 (72)	1.1 (351)	<.0001	2.2 (70)	1.2 (386)	<.0001
Target vessel–related myocardial infarction	1.7 (52)	0.8 (264)	<.0001	1.6 (50)	0.9 (296)	<.001
CD-TVR	2.6 (83)	2.2 (717)	.15	2.6 (83)	2.3 (754)	.32
CD-TLR	1.9 (59)	1.7 (532)	.37	1.9 (59)	1.7 (559)	.61
CD-TV non-TLR	0.9 (29)	0.7 (223)	.15	0.9 (29)	0.7 (238)	.27
Stent thrombosis, definite and probable	0.9 (29)	0.7 (209)	.08	0.9 (28)	0.8 (247)	.41
Major bleeding	1.6 (49)	0.5 (153)	<.0001	1.5 (48)	0.6 (202)	<.0001
Stroke	0.4 (14)	0.1 (45)	<.0001	0.4 (14)	0.2 (51)	<.001

Values are % (n).

The 1-year event rates are based on patients who reached the 1-year follow-up or had a primary end point event.

CD-TLR, clinically driven target lesion revascularization; CD-TVR, clinically driven target vessel revascularization; MACE, major adverse cardiac events; POCE, patient-oriented composite end point; TLF, target lesion failure; TV, target vessel.

Importantly, the rates of revascularization were similar in the 2 groups, for both target vessel revascularization (TVR) (2.6% vs 2.2%, *P* = .320) and TLR (1.9% vs 1.7%, *P* = .61). Stent thrombosis rates were low and similar among both groups (0.9% and 0.7%, *P* = .08). A similar proportion of patients was free of angina symptoms at 1 year (88.7% vs 88.0%, *P* = .95).

### Propensity analysis

**Figure 2:**
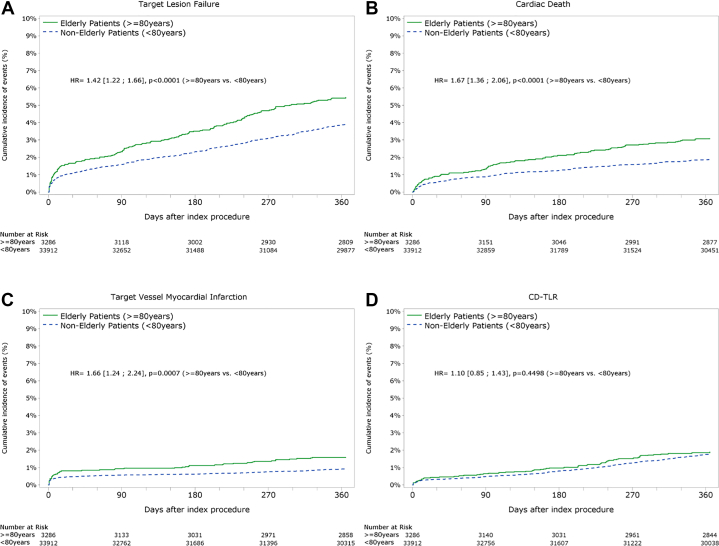
**Outcome after propensity score adjustment.** Kaplan–Meier cumulative incidence of event curves after inverse-weighted propensity score adjustment: target lesion failure (**A**), cardiac death, (**B**) target vessel–related myocardial infarction, (**C**) and clinically driven target lesion revascularization (**D**).

**Figure 3:**
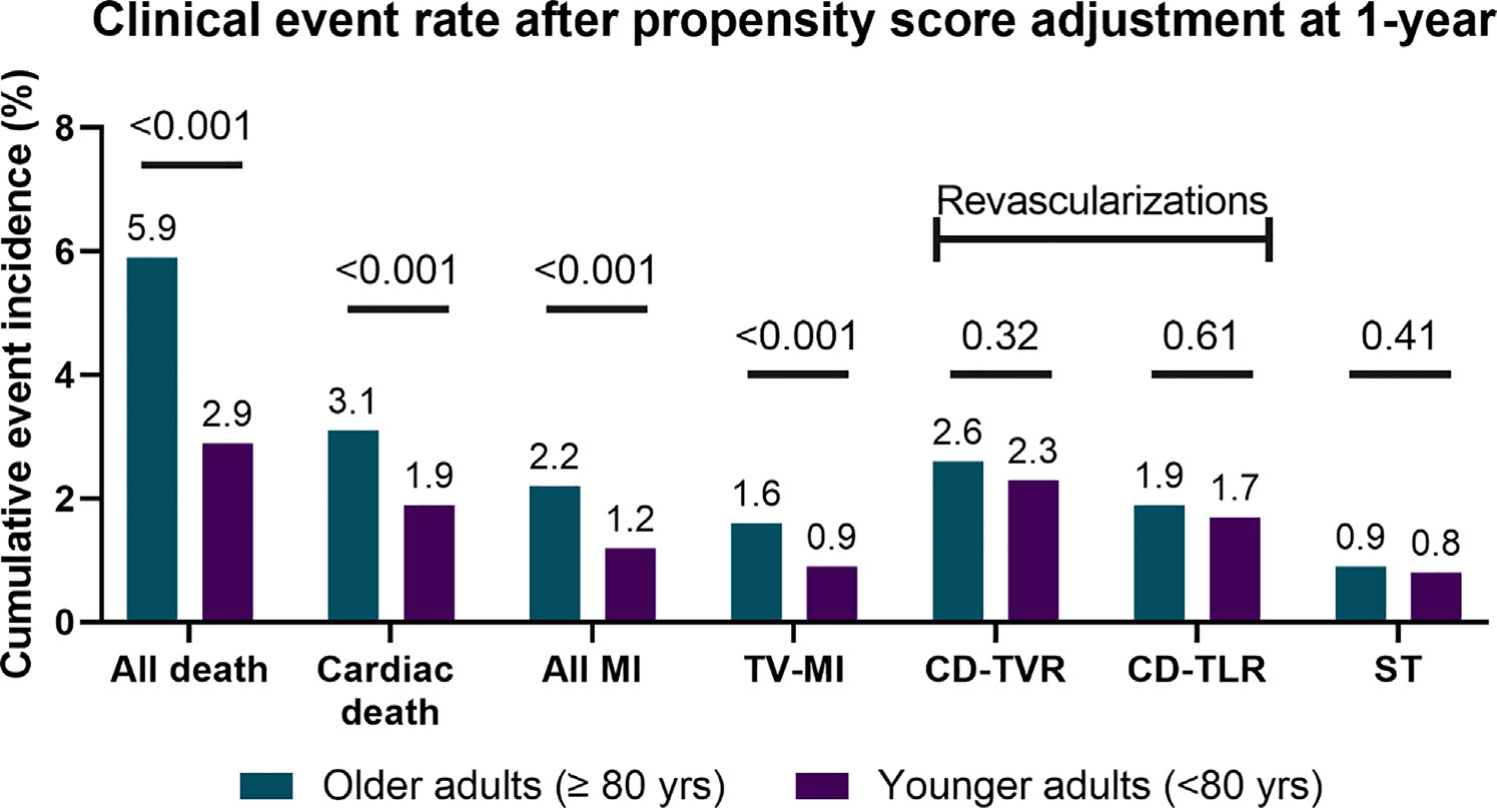
**Cumulative event rates after propensity score adjustment at 1-year showing higher event rates for the older adult group except for the revascularizations.** CD-TVR, clinically driven target vessel revascularization; CD-TLR, clinically driven target lesion revascularization; MI, myocardial infarction; TV-MI, target vessel–related myocardial infarction; ST, stent thrombosis (definite/probable).

After adjustment with the propensity score, older adults aged > 80 years were found to be associated with 42% increased risk of TLF (95% CI, 22-66; *P* < .0001), 67% added risk of cardiac death (95% CI, 36-106; *P* < .0001), and 66% added risk of TV-MI (95% CI, 24-124; *P* < .001), but not for an increased risk of CD-TLR (HR, 1.10; 95% CI, 0.85-1.43; *P* = .45) (Central Illustration, Figures 2 and 3). Even after adjustment, major bleeding was more common in the older adult group (1.6% vs 0.6%, *P* < .0001).

**Central Illustration:**
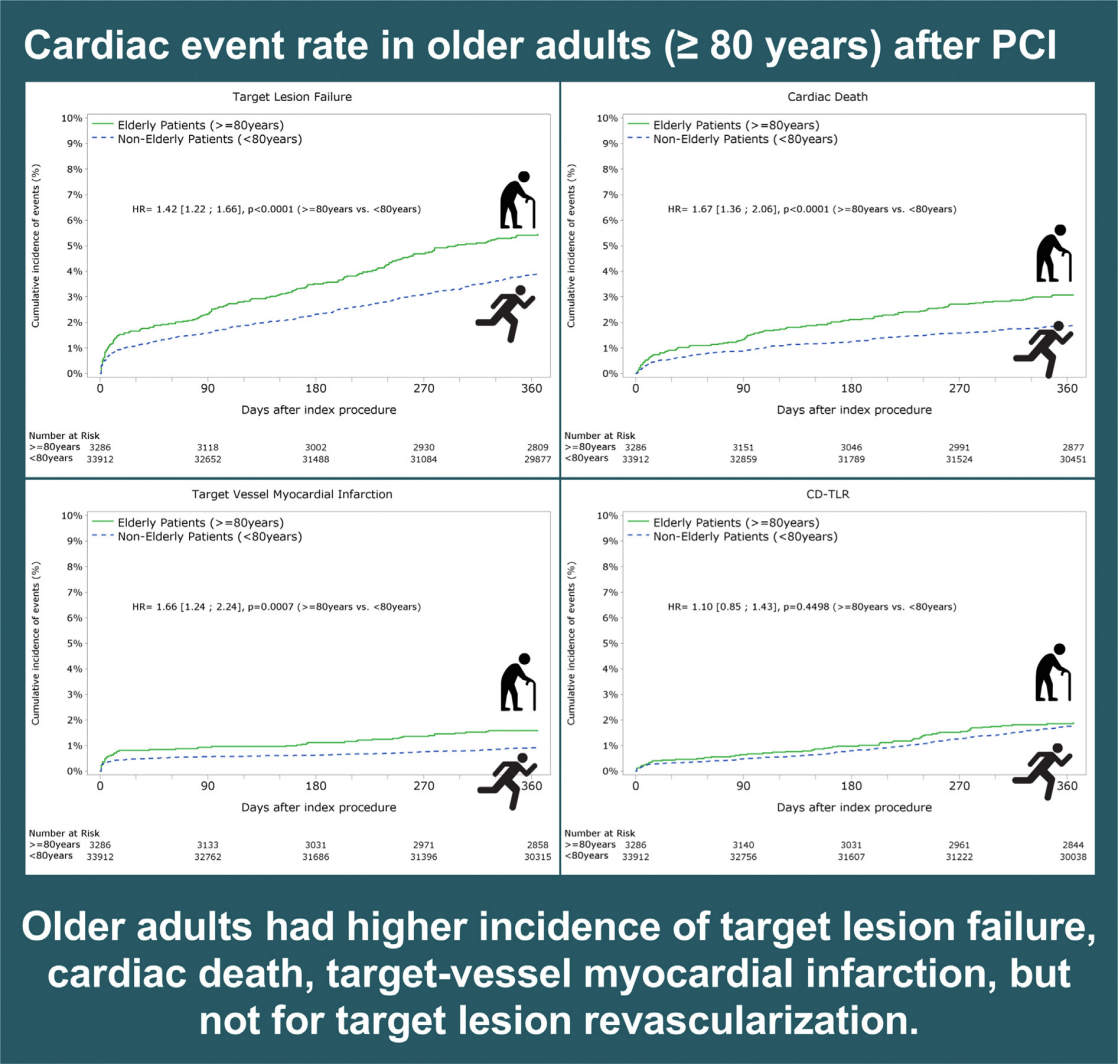
Cardiac event rates in older adults (≥80 years) after PCI. Older adults had a higher incidence of target lesion failure, cardiac death, target vessel myocardial infarction, but not for target lesion revascularization.

## Discussion

We investigated the outcomes after PCI according to age in a large, real-world registry of >37,000 patients. To our knowledge, the current report represents one of the largest analyses of PCI outcomes in all-comers older adult patients who underwent PCI with new-generation thin-strut DES. The crude and adjusted rates of the primary end point of TLF were significantly higher in the older adult group than those in the younger group. Furthermore, secondary outcomes such as all-cause death, cardiac death, and MI rates were significantly higher in the older adults than those in the younger group, as were rates of major bleeding and stroke.

Despite the growing need for PCI among the older adults, there is a paucity of data about outcomes after PCI in this group. This group is frequently excluded from clinical trials, mainly because of concerns about the increased risk of adverse events and limited life expectancy.^11^ Most of the published data on outcomes among the older adults originated from the Japanese population.^16^^,^^18^, ^19^, ^20^ More recently, new randomized trials were published, the SENIOR and XIMA trials that compared DES and bare-metal stents, respectively.^21^^,^^22^ The latest published data from a patient-level pooled analysis of the TWENTE trials included 671 older adult patients compared with 8533 younger patients treated with DES.^23^

In our study, the rate of the primary end point of 1-year TLF was significantly higher in the older age group (5.6% vs 3.0%, *P* < .0001). The proportion of patients with cardiac death (3.3%) and TVR (2.6%) was similar to the rates reported in the DES arm of the randomized XIMA and SENIOR trials.^21^^,^^22^ Moreover, the data from TWENTE I-IV trials showed similar results of cardiac death rates (3.3%), TV-MI (2.3%), and TVR (1.9%).^23^

As the results of our study show, the older adult group who underwent PCI had more complex procedures. Nonetheless, transradial artery intervention rates were similarly high in both older adults and younger groups despite the higher complexity of PCI and higher comorbidity burden in the older adult group, a finding that is consistent with previous studies.^20^^,^^21^ This finding emphasizes the feasibility and the high success rate of radial access in fragile patients. A large Japanese registry has shown that transradial artery intervention was an inverse-independent predictor of both in-hospital mortality and bleeding complications in both acute coronary syndrome and stable CAD cohorts.^16^

Safety is a major concern in the older adult patients. Bleeding complications were more prevalent in the older adult group compared with those in the younger group, with similar rates reported in studies using the new-generation DES in the older adults.^19^, ^20^, ^21^, ^22^, ^23^ The higher incidence in the older adult group may be attributed to the higher prescription of anticoagulant medication and higher prevalence of renal dysfunction found in this group. However, even in the older adult group, the rate of major bleeding was relatively low (1.6% at the 1-year follow-up).

Stent thrombosis is another aspect of postprocedural safety. The low rates of stent thrombosis in our trial are consistent with previous randomized trials with a polymer-free, drug-coated stent in patients at high bleeding risk and with a DES in patients in their 80s,^19^, ^20^, ^21^, ^22^, ^23^, ^24^ establishing the advantages of using the new-generation DES over older stents.

The incidence of TVR and non-TVR was similar between the older adult and the younger patients in both the crude and adjusted analyses as opposed to the other events that showed higher rates in the older adult group. There was no difference in the incidence of symptom-free patients at 1 year. Therefore, it seems unlikely that this is because of a more restricted approach regarding revascularization toward older adult patients. The difference in device and patient-oriented clinical composite outcomes is driven by non–revascularization-related events, and additional research is needed to effectively manage the coronary disease and the comorbidities to further improve the prognosis in older adult patients after PCI.

The analysis and comparison of longer-term mortality rates between the 2 groups require specific consideration. The baseline life expectancy of elderly adults is, needless to say, more limited than that of younger patients. Therefore, a comparison of 1-year morality rate between the groups has limited added value, and the baseline life expectancy must be considered. The 1-year mortality rate of 83-year-old adults (the median age of patients in the older adult group) ranges from 4% to 5.7% for women to 6% to 7.5% for men in the United Kingdom, France, and the United States, whereas the 1-year mortality rate among 62-year-old adults is 0.5% to 0.8% for women and 0.9% to 1% for men.^25^, ^26^, ^27^ A comparison of the mortality rate in our analysis with the expected life expectancy of the general population revealed that older adult patients undergoing PCI have a similar life expectancy (6.2%) to the general population, whereas actually the prognosis of younger patients undergoing PCI (1.7%) is significantly worse than the baseline mortality.

Factors such as frailty, sarcopenia, physical dysfunction, cognitive impairment, and polypharmacy were not measured in our trial. These factors may affect the outcome, especially among the geriatric population.^28^^,^^29^

### Study limitations

Several potential limitations of this study should be noted. First, its observational nature may affect the way these results are translated into a clinical basis. Second, the younger group included patients with a wide range of ages, some of whom were close to the age of the older adults >80 years. In addition, indices of frailty that may affect the clinical outcomes were not collected in this trial. Frailty parameters may vary widely among the older adult population. Moreover, event rates were not adjusted for the competing risk of death, which could overestimate the incidence of the other clinical events. Furthermore, unmeasured confounders may not be accounted for in the inverse propensity score weighted analysis, such as the decision to revascularize vessels of someone at an advanced age, presence of other comorbidities, and differences in health insurance organization. Finally, the findings of this study apply to a specific DES platform and cannot, therefore, be extrapolated to other bioabsorbable or durable polymer DES platforms.

## Conclusions

Older adult patients had more comorbidities and a higher incidence of the composite end point TLF than younger patients. The all-cause death, cardiac mortality, and recurrent MI at 1 year were higher for older adult patients than those for patients <80 years. However, there was no difference in the incidence of recurrent revascularization or stent thrombosis. These findings suggest that PCI in the older adults is relatively safe in the era of contemporary DES using a thin-strut cobalt–chromium sirolimus eluting stent. However, even with contemporary DES technology, the older adult patients experience higher rates of post-PCI adverse events. This should also be considered before the decision making regarding treatment strategies in this age group.

## Figures and Tables

**Supplementary Figure 1:**